# Implementing the Infectious Diseases Society of America Antimicrobial Stewardship Core Curriculum: Survey Results and Real-World Strategies to Guide Fellowship Programs

**DOI:** 10.1093/ofid/ofae542

**Published:** 2024-10-02

**Authors:** Leila S Hojat, Payal K Patel, Dilek Ince, Amy Y Kang, Gary Fong, Kartik Cherabuddi, Priya Nori, Hawra Al Lawati, Erica J Stohs, Cole Beeler, Trevor C Van Schooneveld, Matthew S Lee, Keith W Hamilton, Julie Ann Justo, Jennifer O Spicer, Ashleigh Logan, Kenza Bennani, Rostam Williams, Rachel Shnekendorf, Chloe Bryson-Cahn, Zachary I Willis, Ryan P Moenster, Thea Brennan-Krohn, Molly L Paras, Marisa Holubar, David C Gaston, Sonali D Advani, Vera P Luther

**Affiliations:** Department of Medicine, Case Western Reserve University, Cleveland, Ohio, USA; Department of Internal Medicine, Intermountain Health, Murray, Utah, USA; Department of Internal Medicine, University of Iowa Carver College of Medicine, Iowa City, Iowa, USA; Department of Pharmacy Practice, School of Pharmacy, Chapman University, Irvine, California, USA; Department of Pharmacy Practice, School of Pharmacy, Chapman University, Irvine, California, USA; Department of Medicine, University of Florida, Gainesville, Florida, USA; Department of Medicine, Albert Einstein College of Medicine, Bronx, New York, USA; Department of Medicine, Beth Israel Deaconess Medical Center, Boston, Massachusetts, USA; Department of Internal Medicine, University of Nebraska Medical Center, Omaha, Nebraska, USA; Department of Medicine, Indiana University School of Medicine, Indianapolis, Indiana, USA; Department of Internal Medicine, University of Nebraska Medical Center, Omaha, Nebraska, USA; Department of Medicine, Beth Israel Deaconess Medical Center, Boston, Massachusetts, USA; Department of Medicine, University of Pennsylvania Perelman School of Medicine, Philadelphia, Pennsylvania, USA; Department of Clinical Pharmacy and Outcomes Sciences, University of South Carolina College of Pharmacy, Columbia, South Carolina, USA; Department of Medicine, Emory University School of Medicine, Atlanta, Georgia, USA; Infectious Diseases Society of America, Arlington, Virginia, USA; Infectious Diseases Society of America, Arlington, Virginia, USA; Infectious Diseases Society of America, Arlington, Virginia, USA; Infectious Diseases Society of America, Arlington, Virginia, USA; Department of Medicine, University of Washington School of Medicine, Seattle, Washington, USA; Department of Pediatrics, University of North Carolina School of Medicine, Chapel Hill, North Carolina, USA; Department of Pharmacy Practice, St Louis College of Pharmacy, St Louis, Missouri, USA; Department of Pathology, Beth Israel Deaconess Medical Center, Boston, Massachusetts, USA; Department of Medicine, Harvard Medical School, Boston, Massachusetts, USA; Department of Medicine, Stanford University School of Medicine, Stanford, California, USA; Department of Pathology, Microbiology, and Immunology, Vanderbilt University Medical Center, Nashville, Tennessee, USA; Department of Medicine, Duke University School of Medicine, Durham, North Carolina, USA; Department of Medicine, Wake Forest University School of Medicine, Winston-Salem, North Carolina, USA

**Keywords:** antimicrobial stewardship, curriculum development, infectious diseases fellowship training, medical education, survey

## Abstract

**Background:**

The Infectious Diseases Society of America (IDSA) developed the Core Antimicrobial Stewardship (AS) Curriculum to meet the increasing demand for infectious diseases (ID) providers with AS expertise. Notable diversity in implementation approaches has been observed among ID fellowship programs using the curriculum. We sought to describe individual approaches and develop a curriculum implementation roadmap.

**Methods:**

We surveyed ID fellowship programs that had previously implemented the IDSA Core AS curriculum. The survey included questions regarding program characteristics, curriculum participants and presentation format, resources and barriers, and implementation strategies. Commonly reported program features were summarized in the context of the self-reported implementation strategies. Implementation guides were developed based on the most common characteristics observed.

**Results:**

Of 159 programs that had purchased the curriculum, 37 responded, and 34 (21%) were included in the analysis. The curriculum was primarily taught by AS physicians (85%) and AS pharmacists (47%). The most common conference structure was a longitudinal conference series (32%), and eLearning was the most common presentation format. Limited AS faculty time (76%) and limited first-year fellow availability (62%) were frequently reported as barriers, and dedicated AS curricular time was a resource available to most programs (67%); implementation guides were created for these 3 program features.

**Conclusions:**

Programs reported a variety of implementation barriers and resources, with several common themes emerging, allowing for the development of tailored curriculum planners for 3 commonly observed program characteristics. This work will equip fellowship programs with curriculum implementation strategies and guide future enhancements of the IDSA Core and Advanced AS curricula.

## Teaser

The IDSA Antimicrobial Stewardship Curriculum is designed for infectious diseases fellowship training, but unique program features may warrant a tailored implementation approach. We surveyed program directors to characterize current implementation strategies and offer guides that accommodate common program barriers and resources.

## Graphical Abstract

**Graphical Abstract:**
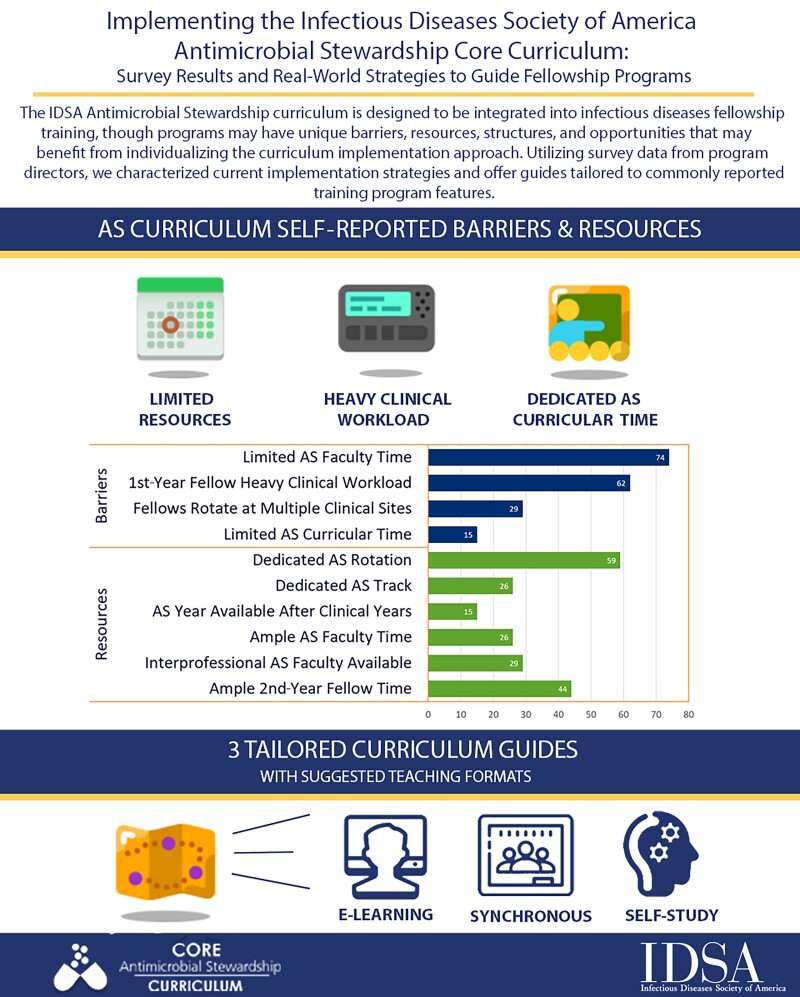

Antimicrobial resistance is a global health threat that has been growing at an alarming rate fueled by the inappropriate use of antibiotics [1–3]. This underscores the necessity to introduce antimicrobial stewardship (AS) concepts early in the training of medical professionals to help combat antimicrobial resistance via judicious prescribing, leading to improved patient outcomes and reduced healthcare costs [4–6]. The Infectious Diseases Society of America (IDSA) developed the Core Antimicrobial Stewardship Curriculum to meet the escalating demand for expertise in this field and in response to an identified need for training materials in AS by infectious diseases (ID) fellowship program directors [7]. The curriculum provides education for healthcare professionals, particularly ID fellows and ID pharmacists, on best practices in AS and operational aspects of stewardship programs. The curriculum has been well-received by ID training programs as demonstrated by a survey showing that implementation increased overall satisfaction with AS training among both ID fellows and program directors. Additionally, the curriculum increased fellows’ confidence and knowledge scores in AS [8].

The IDSA stewardship curriculum provides online access to the curriculum materials, with 1 version formatted for learners and a parallel version of the material formatted for teachers. This method of curriculum delivery offers flexibility for adult learners in training, with opportunities to integrate the teaching material into existing fellowship curricula versus allowing a learner-driven approach combined with additional guidance and context from local AS leaders. Therefore, implementation is heavily dependent on local training programs’ resources and infrastructure. The success of integrating the IDSA stewardship curriculum into diverse clinical environments hinges on adapting to these varying capabilities. Tailoring the implementation approach to suit the specific needs and constraints of each program is crucial for effective learning and application.

To further enhance the reach and impact of this curriculum, we designed a survey to study its current utilization among ID fellowship programs across various healthcare settings. This survey gathered detailed feedback on the curriculum's effectiveness, barriers to implementation, and the diverse approaches taken to integrate the curriculum into training programs, thereby increasing its adaptability and uptake. We used the survey results to develop a strategic tool to assist ID fellowship program directors with curriculum implementation based on individual program resources and barriers.

## METHODS

### Survey Design

We designed a survey for ID fellowship program directors who had previously implemented the core curriculum with their fellows. We focused on ID fellowship programs as these represent over 90% of the curriculum participants and were also the initial target group for this education. The survey included questions related to program characteristics, participants (learners and instructors), format of curriculum presentation, resources and barriers to curriculum implementation, general curriculum satisfaction, and considerations of how implementation of the curriculum could be enhanced. Additionally, several of the questions were focused on implementation of IDSA's Advanced Antimicrobial Stewardship Curriculum, which has been offered separately since July 2021. The survey contained a total of 40 items, including 6 questions utilizing a Likert scale, 4 yes-or-no questions, 20 questions with a list of items to choose from, and 10 questions requiring a free-text response (see Supplementary material for full survey text). Several of the items requiring a selection from available choices also allowed respondents to provide comments. The complete list of survey questions is included in the Supplementary material. The survey was created using Google Forms, and 2 individuals who were not part of the study team tested the survey for ease of use and timing, which was determined to be approximately 10–15 minutes.

### Survey Distribution

A link to the survey was distributed via email to the contact provided for all programs nationally or internationally that had previously purchased the curriculum, and 2 reminder emails were sent over the course of a 3-week period. A chance to be provided free access to an IDSA Academy Maintenance of Certification course was offered as an incentive for completion of the study, though this incentive was made optional in order to preserve anonymity if this were preferred by the study respondent. Participants also could volunteer to provide their contact information if they were willing to receive additional communication from the study team in the future.

### Analysis

Responses were categorized according to program type, and programs not utilizing the curriculum for an ID fellowship program were excluded. Survey responses were analyzed using descriptive statistics. Commonly reported program resources and barriers were summarized in context of strategies programs used for implementation of the curriculum. We used the responses to develop course planners tailored to the most common program features observed in terms of their self-identified barriers to implementation as well as available resources. This study was exempted from review by the University Hospitals of Cleveland Institutional Review Board.

## RESULTS

### Program Characteristics

A total of 159 institutions were identified that had previously purchased the curriculum as of June 2022, and 517 individuals associated with these institutions including program directors and other designated faculty received the survey invitation email. These included 29 institutions that participated during the 2018–2019 pilot year, 42 that purchased the curriculum in 2019, 30 in 2020, 55 in 2021, and 3 in 2022. Three programs were located outside the United States (US). Of the 159 programs, 37 completed the survey, for an overall response rate of 23%. Three institutions used the curricula with non–ID fellow learners only and were excluded. Characteristics of the 34 programs included in the analysis are reported in Table 1. Twenty-four programs (71%) had participated in the curriculum for 2 or more years; of these, 19 (79%) and 5 (21%) used the curriculum every year and every other year, respectively. The most common learners utilizing the curriculum were ID fellows (100%), pharmacy trainees (79%), and ID faculty (74%). Participating programs were of moderate size with a median 3 fellows per year, and many had dedicated AS time built into their general academic curriculum. The majority were adult ID programs (94%) versus pediatric, and of the 21 program directors offering identifying information, all represented US programs.

**Table 1.** Descriptive Characteristics of the 34 Infectious Diseases Fellowship Programs Participating in the Survey

Program Characteristic	Programs, No. (%)^a^
No. of years participated in curriculum	
1	10 (29)
≥2	24 (71)
Academic years participated in curriculum	
2018–2019	13 (38)
2019–2020	15 (44)
2020–2021	18 (53)
2021–2022	16 (47)
Frequency of curriculum implementation^b^	
Every year	19 (79)
Every other year	5 (21)
Curriculum learners	
ID fellows	34 (100)
ID faculty	25 (74)
ID pharmacists	1 (3)
Pharmacy trainees	27 (79)
IM residents	3 (9)
Medical students	6 (18)
Curriculum teachers	
AS program faculty	29 (85)
Interprofessional AS faculty	16 (47)
Other ID faculty	6 (18)
ID fellows	4 (12)
None (self-study)	3 (9)
Dedicated AS curricular time	
AS rotation	20 (59)
AS track	9 (26)
AS postclinical year	5 (15)
Fellows per year, median (IQR)	3 (2–4)

Abbreviations: AS, antimicrobial stewardship; ID, infectious diseases; IM, internal medicine; IQR, interquartile range.

^a^Values are presented as No. (%) except where indicated. Total percentages may not be equal to 100% for characteristics allowing for multiple responses.

^b^Includes 24 programs that participated in the curriculum for 2 or more years.

### Curriculum Presentation

The curriculum was primarily taught by AS physicians (85%) and AS pharmacists (47%), though other non-AS ID faculty members and ID fellows contributed to teaching in a small number of programs (Table 1). The most common presentation structure of the curriculum was through longitudinal conferences (32%), followed by an early “bootcamp” block (24%) or a designated block later in the academic year (24%). The eLearning modules (ie, narrated, interactive slides) were the primary strategies used for presenting the AS content, in both synchronous (ie, teacher and learner reviewing material together) and asynchronous (ie, self-study) contexts, with 62% using the eLearning modules for at least a portion of their module presentations (Table 2). Use of the unguided lecture slides was primarily limited to programs presenting the curriculum as part of a longitudinal conference structure or AS block. Programs that had presented the curriculum during multiple academic years often described modifying the method of implementation over time, including moving to more asynchronous teaching (18%), transitioning some or all sessions to a virtual platform (24%), or presenting the curriculum at a different time during the academic year (18%). The coronavirus disease 2019 (COVID-19) pandemic was noted specifically to have influenced many programs to move to a virtual format. Overall curriculum satisfaction was high, with 91% of programs reporting being “satisfied” or “very satisfied” with the course.

**Table 2.** Implementation Structures and Presentation Formats Utilized Within Each Structure Category

Conference Structure (n = 34)	Synchronous eLearning	Asynchronous eLearning	In-person Unguided Lecture Slides	Virtual Unguided Lecture Slides
Bootcamp (n = 8)	4	4	1	1
Longitudinal conference (n = 11)	7	7	4	5
Academic half day (n = 2)	1	1	0	1
Later block (n = 4)	2	3	0	1
AS block (n = 8)	3	5	2	4
Self-study (n = 1)	0	1	0	0

Conference structure refers to the primary setting reported by each program for curriculum implementation. Multiple presentation formats may have been utilized by programs within each conference structure type.

Abbreviation: AS, antimicrobial stewardship.

### Barriers and Resources

**Figure 1.**
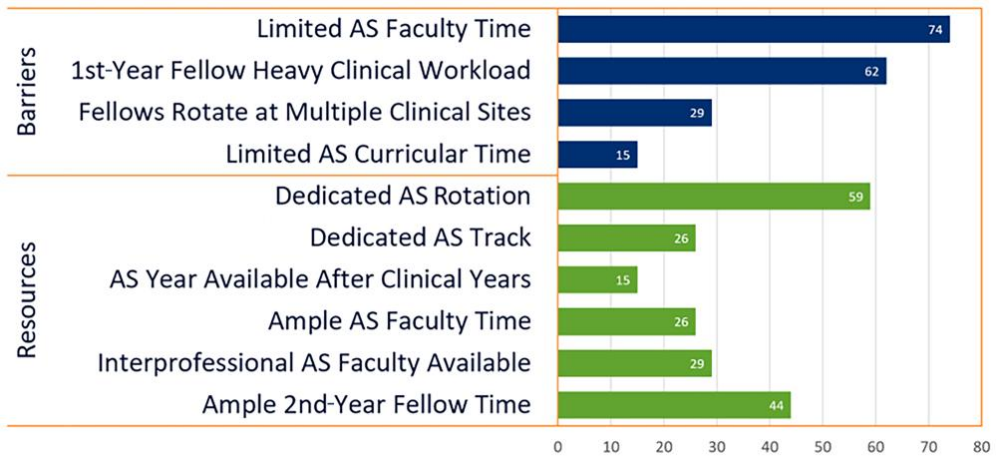
Percentage of total infectious diseases fellowship programs (n = 34) self-reporting identified barriers and resources to effective antimicrobial stewardship (AS) curriculum implementation.

**Figure 2.**
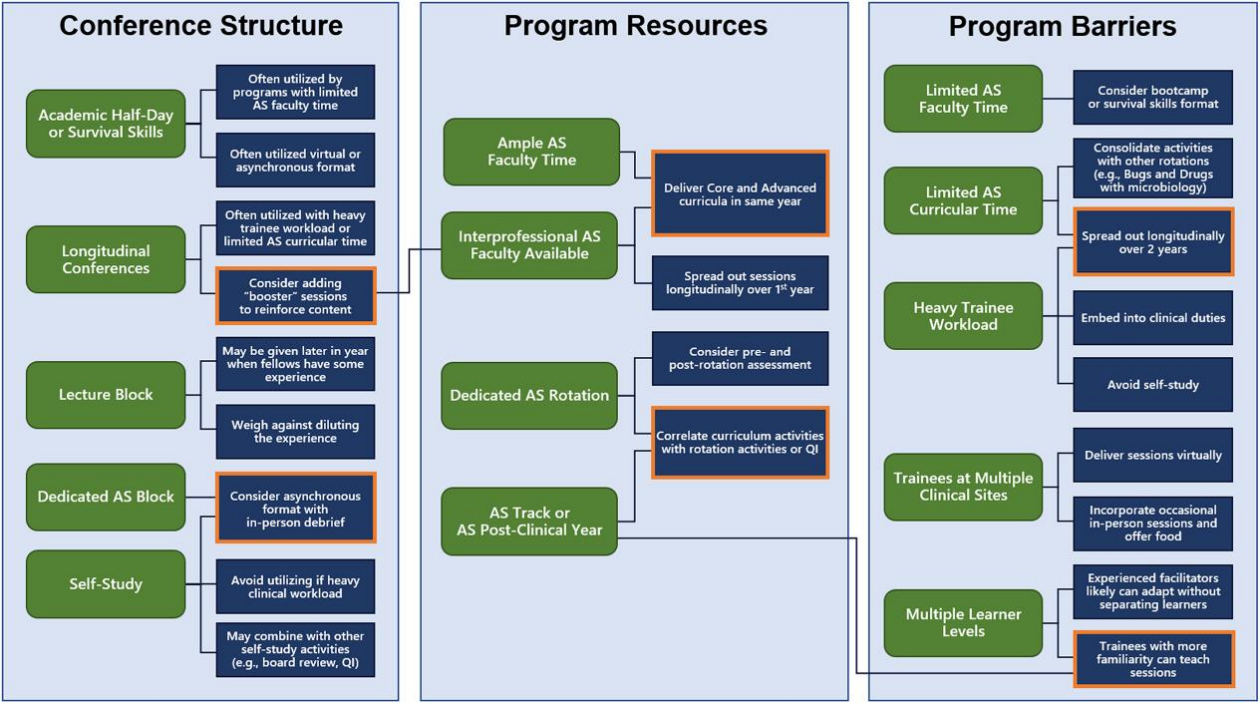
Strategies for curriculum implementation (dark blue rectangles) utilized or suggested by programs based on characteristics of conference structure, resources, and barriers (green rounded rectangles). Highlighted rectangles (orange outline) indicate strategies applied to multiple characteristics. Abbreviations: AS, antimicrobial stewardship; QI, quality improvement.

The most common barrier to implementation of the curriculum was limited AS faculty time (74%); for example, 1 program indicated clinical staff shortages limiting the amount of teaching time available to the AS faculty (Figure 1). Additionally, heavy first-year fellow workload (62%) was a barrier experienced by many programs, with several programs indicating the specific importance of presenting the curriculum early in the first year when the concepts are most pertinent. While 15% of programs indicated limited AS curricular time as a barrier to implementation, 67% of programs (n = 23) reported having an AS rotation and/or track as a resource within their programs (3 reported an AS track only). Additionally, 15% of programs offered an optional year of dedicated AS training after the completion of clinical training. Programs also noted the availability of interprofessional AS faculty such as pharmacy (29%) and ample second-year fellow time (44%) as resources. For each barrier, resource, or conference structure, programs provided suggestions or indicated strategies they had utilized in order to improve the efficiency and effectiveness of curriculum implementation, as summarized in Figure 2.

### Advanced Curriculum

Of the 34 programs, 19 (56%) also implemented the advanced curriculum, with 11 (58%) of these programs doing so in the same calendar year and 9 (47%) implementing both curricula within a dedicated block. Primary learners in the advanced curriculum were second-year fellows (53%), selected fellows with specific interest (37%), and first-year fellows (32%). Fourteen (74%) programs delivered the curriculum asynchronously, and 10 (53%) utilized the eLearning modules.

## DISCUSSION

The IDSA Core Antimicrobial Stewardship Curriculum provides a robust framework for delivery of a challenging topic, while maintaining the flexibility to adapt to a variety of contexts. It is the first formal curriculum that has standardized and provided both the fundamental and advanced AS skills required to train future ID physicians and AS leaders in an area with clear national demand [9]. In this study, we identified characteristics of and barriers to implementation of the core curriculum, furthering our understanding of how the material can be customized to diverse training environments. We used the various strategies described within heterogeneous fellowship program structures to inform the development of tailored curriculum implementation guides, facilitating the sharing of successful tactics between different programs.

This study identified a large degree of variability in curriculum implementation strategies. Implementation of curricular elements took place in longitudinal conference structures, academic half-days, introductory fellowship lectures, set curricular blocks, and as self-study, each with benefits and drawbacks depending on the individual program. Presenting the curriculum in a longitudinal format may pose issues with retention of information discussed in earlier sessions, yet this approach may be more suitable for busier fellowships where service requirements cannot accommodate a dedicated lecture block. Self-study allows curricular access in the setting of limited time available for in-person activities but loses certain benefits from modules better delivered with accompanying interpersonal interaction and real-time mentorship. Programmatic strategies reported in this study help offer mitigation for these shortcomings (Figure 2). For instance, longitudinal conference structure may benefit from interposed reinforcement sessions, and self-study could be occasionally supplemented with in-person debriefs.

Our findings highlighted implementation features or “phenotypes,” which in turn facilitated the development of curricular pathways for the 3 most common patterns observed (Table 3). These included 2 barriers (limited AS teaching resources and high first-year fellow clinical burden) and 1 resource (dedicated AS curricular time). Limited AS faculty time for adequate content delivery was the most frequently reported among the various identified obstacles (Supplementary Table 1). A proposed strategy for programs with limited teacher resources is to utilize synchronous implementation of the Bugs and Drugs primer, followed by self-study of certain modules within other sections. Inviting individuals outside of the core AS faculty team to facilitate sessions is another potential approach, including senior fellows, pharmacists, microbiologists, and non-AS ID faculty, who have been demonstrated to be successful participants in AS education [10–12]. Along similar lines of distributing the teaching load, programs could consider collaborating with one another by having an AS faculty member facilitate a session for fellows at multiple programs, either by remote conferencing or in-person for programs in close proximity. While the pandemic was a major driver of limited faculty resources discussed by survey participants, limited preceptor time is also a reality of understaffing and poor ID match rates, which are ongoing factors in the postpandemic period. Thus, the strategies proposed here are likely to remain essential until significant changes to the ID workforce take place.

**Table 3.** Simplified Curriculum Planner for Programs With Limited Instructor Resources

Module	Suggested Format	Suggested Lead/Timing
Bugs and Drugs		
Foundations of Microbiology	Synchronous	Microbiology leads
Foundations of Pharmacology	Synchronous	Pharmacy leads
Foundations Cases	Synchronous	Senior ID fellow leads
Section 1		
Introduction	Self-study	Complete prior to other sections
Side Effects	Self-study	…
Role of Clinical Lab	Synchronous	Microbiology leads
Section 2		
Curbside Call	Synchronous	Non-AS ID faculty leads
Antibiotic Approval/PAF	Synchronous	Pharmacy leads
Clinic Visit	Synchronous	AS faculty leads
Rapid Diagnostic	Synchronous	Microbiology leads
Section 3		
Deference to Seniority	Synchronous	Non-AS ID faculty leads
Overly Broad/De-escalation	Synchronous	AS faculty leads
Pediatric Telemedicine	Self-study	…
Section 4		
SMART Exercise: Shortage	Synchronous	Pharmacy leads
Regulatory	Synchronous	AS faculty leads

See Supplementary material for full version and notes.

Abbreviations: AS, antimicrobial stewardship; ID, infectious diseases; PAF, prospective audit and feedback; SMART, shortage mitigation of antibiotics in real time.

Another commonly reported barrier was the heavy clinical workload of first-year fellows, limiting their ability to participate in the curriculum. Mitigating efforts for this setting could include interposing the curriculum on predesigned stewardship or other nonclinical rotations; for example, the Foundations of Microbiology module could be reviewed during the microbiology rotation (often a mandatory elective for ID fellows), and Foundations of Pharmacology could be reviewed during the AS rotation if available (Supplementary Table 2). Self-study should be utilized sparingly in this scenario in order to avoid modules not being completed during busy clinical rotations. Finally, a third common ID fellowship phenotype identified was having designated AS time built into the general ID fellow curriculum, which was considered a resource for curriculum implementation. In this setting, certain high-yield modules may be useful to review and reinforce during the AS rotation or track (Supplementary Table 3). Further, AS faculty with dedicated AS curricular time available may also have opportunities to supplement the IDSA curriculum with additional relevant content.

The findings from this study have important implications for ID fellows in training, who have limited exposure to formal AS curricular material [13]. Wasson et al surveyed ID fellows across the US from 2018 to 2019 regarding AS education, finding that most reported little structured AS training but expressed desire for a defined curriculum with mentorship, dedicated time, and formal feedback, regardless of anticipated career choice [14]. The utility of formal AS education is further supported by its observed association with enhanced guideline concordance as well as increased learner confidence and satisfaction with their training [8, 15]. Novel approaches to integrating AS concepts into pharmacy and physician learning should be explored further, such as slowly titrating AS concepts beginning with pregraduate and continuing into postgraduate education or extending AS training into a full dedicated year beyond general ID training; notably, 15% of programs included in our study offered a dedicated AS year [16, 17]. The ID clinical community needs adaptable course content that can be utilized by a variety of learner groups to serve the unmet need for more widespread stewardship recognition and competence.

There are few other published examples of AS curricula targeted toward ID fellows, and most have involved short, intensive training sessions or workshops. In 2013, 2 New York medical centers conducted a half-day AS and infection prevention and control workshop for ID fellows [18]. Almost all participants considered the workshop a valuable supplement to their ID training; notably, over 80% of these participants reported considering a future career involving AS or infection prevention and were actively seeking more training in these areas. Another academic center developed a 6-hour course for ID fellows involving simulated interdisciplinary AS scenarios, and similarly, fellows described high levels of satisfaction with the training [19]. Other available AS educational opportunities include programs offered at national conferences or via online training, though these are designed for a wide range of learners, generally offer limited opportunities for interaction, and likewise tend to be formatted as concentrated sessions. To our knowledge, the IDSA curriculum is the only available AS resource that can be widely adapted to other didactic formats such as longitudinal conferences, interactive sessions, and asynchronous learning.

This study has notable strengths. The principal importance of this work was elucidation of real-world experience utilizing and adapting the IDSA AS curriculum among a wide range of programs, with participants familiar with the curriculum providing a practical approach to its implementation. The comments are directly applicable despite the survey's administration in 2022, as the only major curriculum update since that time was the addition of a pediatric telehealth module in December 2023; the Bugs and Drugs primer was added during the study period in July 2021. Additionally, survey participants provided examples of curriculum adoption across diverse settings and learner and teacher types, shedding light on the delivery of AS curriculum content in multiple contexts. Furthermore, we were able to use the real-world experience in context of a variety of resources and barriers to generate a practical approach or “roadmap” for programs implementing the curriculum in the future, whether for the first time or for optimization of subsequent presentations.

This study also has several limitations. Among the 149–163 adult and 65–66 pediatric US ID programs accredited during the study period, participation in this survey was only performed by those who had already previously purchased the curriculum, limiting its generalizability; however, survey participants represented diverse programs in terms of size and variety of participating instructor and learner types [20]. Another potential limitation is that surveys were targeted toward fellowship program directors who may not have familiarity with all details of how the curriculum was implemented, though there were a few instances in which individuals who had been contacted indicated that they forwarded the survey to their AS leadership for completion. Furthermore, as with most survey study designs, ours is subject to selection bias, and survey participants may represent more committed AS educators. On the other hand, participants may have been more likely to feel that they needed supplemental AS educational resources, in which case they would be well-positioned to provide feedback regarding adaptability of the curriculum [8]. Finally, it is notable that this survey took place in June 2022, at which time pandemic-related constraints may have been ongoing, limiting AS resources and affecting facilitator–learner interactions. While we did not quantitatively analyze how COVID-19 impacted curriculum implementation, several survey participants indicated a switch from in-person to virtual format sessions, and 1 participant acknowledged staffing issues that drew ID specialists away from their primary area of focus [21].

## CONCLUSIONS

We performed a survey of ID fellowship programs participating in the IDSA Antimicrobial Stewardship Core Curriculum and determined that curriculum implementation is highly dependent upon the resources and barriers of individual programs, particularly regarding AS resources and clinical burden on learners. We presented a variety of mitigation strategies based on participant feedback to assist with implementation in specific contexts, including the formulation of tailored implementation guides for the most commonly observed program characteristics. Our study provides practical, peer-to-peer direction for ID fellowship program directors and AS faculty leaders implementing the curriculum within their own institutions. Moving forward, our findings will also inform future enhancements of the IDSA Antimicrobial Stewardship curricula.

## Supplementary Material

ofae542_Supplementary_Data

## Supplementary Data

Supplementary materials are available at *Open Forum Infectious Diseases* online. Consisting of data provided by the authors to benefit the reader, the posted materials are not copyedited and are the sole responsibility of the authors, so questions or comments should be addressed to the corresponding author.

## Notes

***Author contributions*.** L. S. H.: Conceptualization, methodology, investigation, formal analysis, writing–original draft, writing–review and editing, visualization. P. K. P.: Data curation, writing–original draft, project administration. D. I.: Data curation, writing–original draft, visualization. A. Y. K., G. F., and H. A.: Data curation, writing–original draft, writing–review and editing.

K. C., E. J. S., and C. B.: Data curation, writing–original draft. P. N.: Writing–original draft.

T. C. V. and M. S. L.: Data curation, writing–review and editing. K. W. H. and D. C. G.: Writing–review and editing. J. A. J., C. B.-C., Z. I. W., R. P. M., T. B.-K., M. L. P., M. H., and S. D. A.: Data curation. J. O. S.: Methodology. A. L. and R. S.: Project administration. K. B.: Resources, project administration, funding acquisition. R. W.: Resources. V. P. L.: Conceptualization, methodology, writing–review and editing, supervision.

***Acknowledgments.*** The authors thank the Infectious Diseases Society of America for their support of this project including Maintenance of Certification course discounts, staff support, and promotion during the IDWeek conference.

***Data availability.*** Data are not publicly available.

***Patient consent.*** This study does not include factors necessitating patient consent.

***Financial support.*** The authors received no financial support for the research, authorship, and/or publication of this article. Funding for the survey incentive was provided by the Infectious Diseases Society of America in the form of a course discount.
