# Supplementary material for: Implementing the Infectious Diseases Society of America Antimicrobial Stewardship Core Curriculum: Survey Results and Real-World Strategies to Guide Fellowship Programs
Source: Open Forum Infect Dis. 2024 Oct 2;11(10):ofae542. doi: 10.1093/ofid/ofae542 (PMC11450624; doi:10.1093/ofid/ofae542)
Supplement: ofae542_Supplementary_Data [file ofae542_supplementary_data.docx]

**Supplemental Material**: Suggested curriculum planners with key strategies by key program feature (pages S2-S5) and survey materials (pages S6-S15)**.**

**Page S2 – Supplemental Table 1**. Program Feature 1: Limited Teacher Resources.

Key strategies:

- Designate teaching suggested modules to individuals other than AS faculty where available, including microbiology team, pharmacists, non-first-year ID fellows, and non-AS ID faculty.
- Certain sections may be designated for self-study.
- Synchronous format suggested to be presented using eLearning when AS faculty not leading in order to ensure important content is preserved.
- eLearning or unguided slides may be used per user preference for modules led by AS faculty.

**Page S3 – Supplemental Table 2**. Program Feature 2: Limited First-Year Fellow Availability.

Key strategies:

- Avoid self-study.
- Utilize unguided slides where available in order to move through content more efficiently.
- Suggested modules may be deferred to the designated AS rotation if available.
- Suggested modules may be deferred altogether if no ability to include in curriculum and fellows not able to complete as self-study.

**Page S4 – Supplemental Table 3**. Program Feature 3: Dedicated Antimicrobial Stewardship Didactic Time Designated within General Infectious Diseases Fellowship Curriculum.

Key strategies:

- Most content can be presented synchronously during designated conference time.
- Suggested high-yield modules may be helpful to review again and reinforce during the designated AS curricular time.
- Where both formats are available, eLearning or unguided slides may be used per user preference.

**Page S5 – Supplemental Table 4**. Blank curriculum planner.

**Pages S6-S15 – Survey Materials**:

- Page S6 – Initial invitation email
- Page S7 – Survey welcome screen text
- Pages S9-S15 – Survey questions

**Supplemental Table 1**. Program Feature 1: Limited Teacher Resources.

| **Module** | **Suggested Format** | **Suggested Lead/ Timing** |
| --- | --- | --- |
| Bugs and Drugs |  |  |
| Foundations of Microbiology | Synchronous | Microbiology leads |
| Foundations of Pharmacology | Synchronous | Pharmacy leads |
| Foundations: Case One | Synchronous | Senior ID fellow leads |
| Foundations: Case Two | Synchronous | Senior ID fellow leads |
| Foundations: Case Three | Synchronous | Senior ID fellow leads |
| Foundations: Case Four | Synchronous | Senior ID fellow leads |
| Foundations: Case Five | Synchronous | Senior ID fellow leads |
| Section 1 |  |  |
| Introduction | Self-study | Complete prior to other sections |
| Side Effects | Self-study |  |
| Role of Clinical Lab | Synchronous - eLearning | Microbiology leads |
| Section 2 |  |  |
| Curbside Call | Synchronous/ Self-study | Non-AS ID faculty leads |
| Antibiotic Approval | Synchronous | Pharmacy leads |
| Prospective Audit and Feedback | Synchronous | Pharmacy leads |
| Clinic Visit | Synchronous | AS faculty leads |
| Rapid Diagnostic | Synchronous/ Self-study | Microbiology leads |
| Section 3 |  |  |
| Deference to Seniority | Synchronous - eLearning | Non-AS ID faculty leads |
| Overly Broad Empiricism | Synchronous – eLearning or unguided | AS faculty leads |
| De-escalation | Synchronous – eLearning or unguided | AS faculty leads |
| Pediatric Telemedicine | Self-study |  |
| Section 4 |  |  |
| SMART Exercise: Shortage | Synchronous | Pharmacy leads |
| SMARTcase: IDWeek 2018 | Self-study |  |
| Regulatory | Synchronous | AS faculty leads |

Abbreviations: ID, infectious diseases; AS, antimicrobial stewardship; SMART, shortage mitigation of antibiotics in real time.

**Supplemental Table 2**. Program Feature 2: Limited First-Year Fellow Availability.

| **Module** | **Suggested Format** | **Suggested Lead/ Timing** |
| --- | --- | --- |
| Bugs and Drugs |  |  |
| Foundations of Microbiology | Defer | Review during micro rotation |
| Foundations of Pharmacology | Synchronous/ Defer | Review during AS rotation |
| Foundations: Case One | Defer | Review during micro rotation |
| Foundations: Case Two | Synchronous |  |
| Foundations: Case Three | Synchronous |  |
| Foundations: Case Four | Defer | Review during micro rotation |
| Foundations: Case Five | Synchronous |  |
| Section 1 |  |  |
| Introduction | Synchronous – unguided slides |  |
| Side Effects | Synchronous – unguided slides |  |
| Role of Clinical Lab | Synchronous – unguided slides |  |
| Section 2 |  |  |
| Curbside Call | Self-study/ Defer | Review during AS rotation |
| Antibiotic Approval | Synchronous | Focus on duration of therapy |
| Prospective Audit and Feedback | Synchronous |  |
| Clinic Visit | Self-study/ Defer | Review during AS rotation |
| Rapid Diagnostic | Synchronous |  |
| Section 3 |  |  |
| Deference to Seniority | Synchronous – unguided slides |  |
| Overly Broad Empiricism | Synchronous – unguided slides |  |
| De-escalation | Synchronous – unguided slides |  |
| Pediatric Telemedicine | Self-study/ Defer | Review during AS rotation |
| Section 4 |  |  |
| SMART Exercise: Shortage | Self-study/ Defer | Review during AS rotation |
| SMARTcase: IDWeek 2018 | Self-study/ Defer | Review during AS rotation |
| Regulatory | Synchronous |  |

Abbreviations: ID, infectious diseases; AS, antimicrobial stewardship; SMART, shortage mitigation of antibiotics in real time.

**Supplemental Table 3**. Program Feature 3: Antimicrobial Stewardship Didactic Time Designated within General Infectious Diseases Fellowship Curriculum

| **Module** | **Suggested Format** | **Suggested Lead/ Timing** |
| --- | --- | --- |
| Bugs and Drugs |  |  |
| Foundations of Microbiology | Synchronous | Review content during microbiology rotation |
| Foundations of Pharmacology | Synchronous | Review during AS rotation |
| Foundations: Case One | Synchronous | Review content during microbiology rotation |
| Foundations: Case Two | Synchronous | Review during AS rotation |
| Foundations: Case Three | Synchronous | Review during AS rotation |
| Foundations: Case Four | Synchronous | Review content during microbiology rotation |
| Foundations: Case Five | Synchronous | Review during AS rotation |
| Section 1 |  |  |
| Introduction | Synchronous/ Self-study |  |
| Side Effects | Synchronous/ Self-study |  |
| Role of Clinical Lab | Synchronous – eLearning or unguided | Review content during microbiology rotation |
| Section 2 |  |  |
| Curbside Call | Synchronous |  |
| Antibiotic Approval | Synchronous | Review during AS rotation |
| Prospective Audit and Feedback | Synchronous | Review during AS rotation |
| Clinic Visit | Synchronous |  |
| Rapid Diagnostic | Synchronous |  |
| Section 3 |  |  |
| Deference to Seniority | Synchronous – eLearning or unguided | Review during AS rotation |
| Overly Broad Empiricism | Synchronous – eLearning or unguided | Review during AS rotation |
| De-escalation | Synchronous – eLearning or unguided | Review during AS rotation |
| Pediatric Telemedicine | Synchronous/ Self-study |  |
| Section 4 |  |  |
| SMART Exercise: Shortage | Synchronous | Review during AS rotation |
| SMARTcase: IDWeek 2018 | Synchronous/ Self-study |  |
| Regulatory | Synchronous | Review during AS rotation |

Abbreviations: ID, infectious diseases; AS, antimicrobial stewardship; SMART, shortage mitigation of antibiotics in real time.

**Supplemental Table 4**. Blank curriculum planner to allow for customization of curriculum presentation approach.

| **Module** | **Format** | **Lead/ Timing** |
| --- | --- | --- |
| Bugs and Drugs |  |  |
| Foundations of Microbiology |  |  |
| Foundations of Pharmacology |  |  |
| Foundations: Case One |  |  |
| Foundations: Case Two |  |  |
| Foundations: Case Three |  |  |
| Foundations: Case Four |  |  |
| Foundations: Case Five |  |  |
| Section 1 |  |  |
| Introduction |  |  |
| Side Effects |  |  |
| Role of Clinical Lab |  |  |
| Section 2 |  |  |
| Curbside Call |  |  |
| Antibiotic Approval |  |  |
| Prospective Audit and Feedback |  |  |
| Clinic Visit |  |  |
| Rapid Diagnostic |  |  |
| Section 3 |  |  |
| Deference to Seniority |  |  |
| Overly Broad Empiricism |  |  |
| De-escalation |  |  |
| Pediatric Telemedicine |  |  |
| Section 4 |  |  |
| SMART Exercise: Shortage |  |  |
| SMARTcase: IDWeek 2018 |  |  |
| Regulatory |  |  |

Abbreviations: SMART, shortage mitigation of antibiotics in real time.

**Survey Invitation Email**:

*Email subject line:* Invitation to participate in IDSA Antimicrobial Stewardship Curriculum implementation survey

*Email text:*

Dear ID Program Directors and Stewardship Faculty,

You are receiving this invitation as a faculty member who has implemented the **IDSA Antimicrobial Stewardship Curriculum** in some capacity. We are seeking to learn from **your experiences** in using the curriculum, understanding that each program has attributes which serve as both resources and limitations with regard to stewardship activities. We aim to use this information to **enhance the experience** of all those who seek to use IDSA Antimicrobial Stewardship Curriculum in the future.

A research team at University Hospitals of Cleveland/Case Western Reserve University School of Medicine and Wake Forest University School of Medicine is currently seeking participants to join a **survey study**. The purpose of this study is to establish a comprehensive description of how the IDSA Antimicrobial Stewardship Curriculum is being implemented by individual institutions, focusing on **utilization of the Core Curriculum for infectious diseases fellows**. We hope to identify barriers to effective program integration as well as potential solutions. This information will ultimately be used to develop more **targeted guidance to assist program directors** with successful curriculum implementation.

To thank you for your participation, all respondents will be entered to win a **free IDSA Academy MOC course**!

We will send up to 2 reminders about this study. If you would prefer not to be contacted for this study or have any questions about the research, please contact Leila Hojat [email address] or Kenza Bennani [email address; phone number].

This research has been approved by the Institutional Review Board of University Hospitals, which is charged with protecting the rights and welfare of people who take part in research studies. The content of this message has been approved by the UH IRB.

**Please click the link to the survey below to participate by June 17, 2022:** Survey Link [link embedded]

If the link above does not work, try copying the link below into your web browser: [survey link URL]

This link should not be forwarded to others. If you believe another person would be better able to complete the survey, please contact Leila Hojat [email address].

**Survey Welcome Screen:**

IDSA Antimicrobial Stewardship Curriculum Implementation Survey

Introduction/Purpose
Welcome to the IDSA Antimicrobial Stewardship Curriculum Implementation Survey!

The following survey questions are designed to establish a comprehensive description of how the curricula are being utilized by individual institutions. The primary focus of this study is to describe the implementation of the Core Curriculum for infectious diseases fellows, but questions regarding other topics are included as well. This information will ultimately be used to develop more targeted guidance to assist program directors with successful curriculum implementation.

Please review the following information and make your selection at the bottom of the form regarding consent. The survey will take approximately 10-15 minutes to complete. Thank you for your participation!

Participation
Your participation in this survey is voluntary. You may refuse to take part in the research or exit the survey at any time without penalty. Some questions are required for inclusion in the study, while you are free to decline other questions if you do not wish to answer for any reason.

Benefits
There will be no direct benefit to you by your participation in this research study. Your participation in this study will help us to identify barriers to efficient and effective program integration and potential solutions. We hope that the knowledge gained will help benefit program directors like you in the future. However, participation in the study will enable you to be entered to win a free IDSA Academy Maintenance of Certification course.

Risks
There is no physical risk to being included in this study. There is a potential risk that your information may be accessed by someone who should not have access, however, we will work to prevent this by following the plan laid out in the confidentiality section below.

Alternatives to Study Participation
The alternative to participation in this study is to NOT participate. This will not affect you or your program in any way.

Financial Information
Your participation in this study will not involve cost to you, and you will not be paid for participation. However, you will be eligible for the incentives described above if you choose to participate.

Confidentiality
We will work to protect your confidentiality by keeping the survey anonymous and by not including identifying questions. Your name or your program name will not be linked to anything you say in the text of any presentations or publications generated by this research. If you choose to provide us with your email address in the event that we may have follow-up questions or to be entered into the drawing, your responses will remain confidential to those outside the research team. Your survey answers will be collected within a Google form where data will be stored in a password protected electronic format. Your responses will only be linked to your email if you choose to provide us with that information. We will remove this information if files are to be shared with other researchers so that no one will be able to identify you from the information we share.

Summary of your rights as a participant in a research study
Your participation in this research study is voluntary. Refusing to participate will not involve any penalty to your program. You may withdraw prior to submitting the survey at any time and for any reason without penalty. Deidentified data from this study may be published, presented, or otherwise made publicly available. If this happens, your identify will not be revealed.

Contact Information
If you have questions at any time about the study or the procedures, you may contact Leila Hojat via email at leila.hojat@UHhospitals.org or Kenza Bennani at 410-697-2054 or via email at [kbennani@idsociety.org](mailto:kbennani@idsociety.org). If you have any questions, concerns, or complaints about the study in the future, you may also contact them later. If the researchers or if you would like to talk to someone other than the researchers about concerns regarding the study, research participant’s rights, or other issues, please call the University Hospitals Cleveland Medical Center’s Research Subject Rights phone line at (216) 983-4979 or write to: The Associate Chief Scientific Officer, The Center for Clinical Research, University Hospitals Cleveland Medical Center, 11100 Euclid Avenue, Lakeside 1400, Cleveland, Ohio, 44106-7061.

**Survey Questions**:

Please select the Agree option to indicate that you have read the above information and have voluntarily agreed to participate in this survey.

- Agree
- Disagree

What is your overall level of satisfaction with the IDSA Antimicrobial Stewardship Core Curriculum?

- Very satisfied
- Satisfied
- Neutral
- Unsatisfied
- Very unsatisfied

During how many academic years has the Core Curriculum been implemented in your program?

- One year
- Two or more years

How frequently is the Core Curriculum being implemented?

- Every year
- Every 2 years
- Every 3 years or longer
- Other (must specify)

During which academic years has the Core Curriculum has been implemented? (Check all that apply.)

- Fall 2018 – Spring 2019

- Fall 2019 – Spring 2020

- Fall 2020 – Spring 2021

- Fall 2021 – Spring 2022

Please confirm that infectious diseases fellows participate as learners in the Core Curriculum.

- Yes, infectious diseases fellows participate in the Core Curriculum

- No, infectious diseases fellows do not participate in the Core Curriculum

In addition to infectious diseases fellows, which learners have participated in the Core Curriculum? (Check all that apply.)

- Infectious diseases faculty

- PGY-2 infectious diseases pharmacy residents

- PGY-1 or non-infectious diseases PGY-2 pharmacy residents

- Pharmacy students

- Pharmacy fellows

- Medical students

- Internal medicine residents

- Other (must specify)

Who has taught the Core Curriculum? (Check all that apply.)

- Infectious diseases antimicrobial stewardship faculty

- Infectious diseases antimicrobial stewardship pharmacy staff

- Other faculty

- Other pharmacy staff

- Infectious diseases fellows

- None/independent learning

- Other (must specify)

In which format was the Core Curriculum presented?

- Consolidated block early in the year (i.e., bootcamp)

- Integrated into longitudinal lecture series with 1 hour or shorter sessions (e.g., noon conference)

- Integrated into longitudinal lecture series with greater than 1 hour sessions (e.g., academic half-day)

- Other (must specify)

In which other formats has the Core Curriculum been presented? (Check all that apply.)

- Consolidated block early in the year (i.e., bootcamp)

- Integrated into longitudinal lecture series with 1 hour or shorter sessions (e.g., noon conference)

- Integrated into longitudinal lecture series with greater than 1 hour sessions (e.g., academic half-day)

- Other (must specify)

Rate your level of agreement with the statement "The curriculum material has been presented exclusively using the e-learning narrated slides, and the non-narrated slides have never or rarely been utilized"

- Strongly agree

- Agree

- Neutral

- Disagree

- Strongly disagree

Rate your level of agreement with the statement "The supplementary handouts and resources have been utilized extensively"

- Strongly agree

- Agree

- Neutral

- Disagree

- Strongly disagree

When did the learners complete their coursework related to the Core Curriculum?*

- Only during sessions

- Both during and prior to or between sessions

- The learners completed all coursework asynchronously

- Unsure

Over the course of the curriculum, how many total hours did the fellows spend completing their coursework outside of sessions?

- < 1 hour

- 1 to 3 hours

- 3 to 5 hours

- > 5 hours

- Unsure

- Not applicable (no coursework completed between sessions)

Did the learners complete their coursework prior to or between sessions successfully?

- Yes

- No

- Partial completion

- Unsure

- Not applicable (no coursework completed between sessions)

Which sections of the Core Curriculum have been implemented? (Check all that apply.)

- Section 1: Introduction to Antimicrobial Stewardship

- Section 2: Antimicrobial Stewardship in Everyday Practice

- Section 3: Educating and Coaching on Antimicrobial Stewardship

- Section 4: Antimicrobial Stewardship Program Logistics

- Bugs and Drugs Primer

How has each section of the Core Curriculum been implemented? (Check all that apply.) [Section 1: Introduction to Antimicrobial Stewardship]

- Asynchronous e-learning

- Synchronous e-learning (in-person or virtual)

- In-person lectures using unguided presentation slides

- Virtual lectures using unguided presentation slides

How has each section of the Core Curriculum been implemented? (Check all that apply.) [Section 2: Antimicrobial Stewardship in Everyday Practice]

- Asynchronous e-learning

- Synchronous e-learning (in-person or virtual)

- In-person lectures using unguided presentation slides

- Virtual lectures using unguided presentation slides

How has each section of the Core Curriculum been implemented? (Check all that apply.) [Section 3: Educating and Coaching on Antimicrobial Stewardship]

- Asynchronous e-learning

- Synchronous e-learning (in-person or virtual)

- In-person lectures using unguided presentation slides

- Virtual lectures using unguided presentation slides

How has each section of the Core Curriculum been implemented? (Check all that apply.) [Section 4: Antimicrobial Stewardship Program Logistics]

- Asynchronous e-learning

- Synchronous e-learning (in-person or virtual)

- In-person lectures using unguided presentation slides

- Virtual lectures using unguided presentation slides

How has each section of the Core Curriculum been implemented? (Check all that apply.) [Bugs and Drugs Primer]

- Asynchronous e-learning

- Synchronous e-learning (in-person or virtual)

- In-person lectures using unguided presentation slides

- Virtual lectures using unguided presentation slides

How has the method of implementation changed over time since the Core Curriculum was originally implemented? (Check all that apply.)

- More asynchronous e-learning

- Curriculum material accessed more frequently

- Curriculum material accessed less frequently

- Some or all sessions transitioned to a virtual platform

- Curriculum offered during a different time within academic year

- No significant changes have occurred

- Other (must specify)

Please elaborate on the rationale for any changes made in the delivery of the Core Curriculum. Consider both COVID and non-COVID related aspects.

(Free text)

With regard to CONFERENCE STRUCTURE, which of the following describe your program in terms of resources and barriers affecting implementation of the Core Curriculum? (Check all that apply.)

- Lecture series presented during 1 hour or shorter blocks

- Academic half-day (i.e., lectures concentrated into 2-hour or longer sessions)

- Early dedicated lecture series for first-year fellows (e.g., bootcamp, survival skills, orientation series)

- Multidisciplinary conferences or workshops

- Sessions contain learners with varying levels of expertise

- Sessions contain a small number of learners

- Other (must specify)

With regard to FACULTY AVAILABILITY, which of the following describe your program in terms of resources and barriers affecting implementation of the Core Curriculum? (Check all that apply.)

- Ample infectious diseases faculty time and expertise available to teach the Core Curriculum

- Limited infectious diseases faculty time available to teach the Core Curriculum

- Limited infectious diseases faculty expertise

- Additional non-infectious diseases faculty available to teach the Core Curriculum (e.g., pharmacy)

- Other (must specify)

With regard to FELLOWSHIP TRAINING, which of the following describe your program in terms of resources and barriers affecting implementation of the Core Curriculum? (Check all that apply.)

- Less curricular time available for teaching than recommended by the Core Curriculum

- Heavy clinical workload for first-year fellows which limits full participation

- Ample time available for second-year fellows to participate

- Fellows train at multiple clinical sites

- Antimicrobial stewardship rotation is offered

- Antimicrobial stewardship education track is offered

- Dedicated antimicrobial stewardship training year beyond the required training period

- Other (must specify)

What are additional barriers to implementation of the Core Curriculum?

(Free text)

What are potential factors which would assist with implementation of the Core Curriculum (e.g., webinars, demonstrations, course planning, etc.)?

(Free text)

How likely is the Bugs and Drugs Primer to be utilized in the next 1-2 years (if not already being utilized)?

- Very likely

- Somewhat likely

- Not likely

- This resources has already been utilized

- I am not aware of this resource

Have you implemented or piloted the Advanced Antimicrobial Stewardship Curriculum?

- Yes

- No

Which learners have participated in the Advanced Curriculum? (Check all that apply.)

- All first-year infectious diseases fellows

- All second-year infectious diseases fellows

- Selected infectious diseases fellows

- Pharmacy residents or fellows

- Internal medicine residents

- Medical students

- Pharmacy students

- Infectious diseases faculty

- Other (must specify)

For infectious diseases fellows who participated in the Advanced Curriculum, during which point in their fellowship was the Advanced Curriculum delivered?

- During first year, concentrated in one block

- During first year, extended longitudinally over the course of several blocks

- During second year, concentrated in one block

- During second year, extended longitudinally over the course of several blocks

- During third or later years, concentrated in one block

- During third or later years, extended longitudinally over the course of several blocks

- Other (must specify)

How has each section of the Advanced Curriculum been implemented? (Check all that apply.) [Section 1: Antimicrobial Stewardship Development and Logistics]

- Asynchronous e-learning

- Synchronous e-learning (in-person or virtual)

- In-person lectures using unguided presentation slides

- Virtual lectures using unguided presentation slides

How has each section of the Advanced Curriculum been implemented? (Check all that apply.) [Section 2: Antimicrobial Stewardship Operations]

- Asynchronous e-learning

- Synchronous e-learning (in-person or virtual)

- In-person lectures using unguided presentation slides

- Virtual lectures using unguided presentation slides

How has each section of the Advanced Curriculum been implemented? (Check all that apply.) [Section 3: Persuasive Communication in Stewardship]

- Asynchronous e-learning

- Synchronous e-learning (in-person or virtual)

- In-person lectures using unguided presentation slides

- Virtual lectures using unguided presentation slides

How has each section of the Advanced Curriculum been implemented? (Check all that apply.) [Section 4: Specialty Stewardship]

- Asynchronous e-learning

- Synchronous e-learning (in-person or virtual)

- In-person lectures using unguided presentation slides

- Virtual lectures using unguided presentation slides

How has each section of the Advanced Curriculum been implemented? (Check all that apply.) [Section 5: Antimicrobial Stewardship Resources and Professional Development]

- Asynchronous e-learning

- Synchronous e-learning (in-person or virtual)

- In-person lectures using unguided presentation slides

- Virtual lectures using unguided presentation slides

Has the Advanced Curriculum been offered within the same academic year as the Core Curriculum?

- Yes

- No

Which strategies were utilized to allow for both curricula to be administered within the same academic year? (Check all that apply.)

- Dedicated block for the Advanced Curriculum

- Advanced fellows assisting with delivery of the Core Curriculum

- Independent study with mentor support for the Advanced Curriculum

- Not applicable; curricula not administered within the same academic year

- Other (must specify)

What challenges have been met during implementation of the Advanced Curriculum?

(Free text)

What are potential factors which would assist with implementation of the Advanced Curriculum (e.g., webinars, demonstrations, course planning, etc.)?

(Free text)

Please indicate your fellowship program type.

- Adult infectious diseases

- Pediatric infectious diseases

Please specify the number of fellow positions per year available in your program.

(Free text)

Rate your level of agreement with the statement "The IDSA Antimicrobial Stewardship Curriculum section of the IDSA Academy website has an intuitive and easy to navigate user interface"

- Strongly agree

- Agree

- Neutral

- Disagree

- Strongly disagree

Please describe any specific issues regarding the website interface.

(Free text)

Please provide any additional comments regarding your program's implementation of the IDSA Antimicrobial Stewardship Curriculum.

(Free text)

Please provide your email address if you would be willing to be contacted for follow-up questions related to your response and if you would like to be entered into the drawing for a free IDSA Academy MOC course.

(Free text)
